# Na^+^/K^+^-ATPase in the lacrimal glands of rabbits and its changes during induced autoimmune dacryoadenitis

**Published:** 2011-09-01

**Authors:** Chuanqing Ding, Michael Lu, Jianyan Huang

**Affiliations:** Department of Cell and Neurobiology, Keck School of Medicine, University of Southern California, Los Angeles, CA

## Abstract

**Purpose:**

To test the hypothesis that expression of Na^+^/K^+^-ATPase subunits in the lacrimal glands (LGs) of rabbits with induced autoimmune dacryoadenitis (IAD) changes.

**Methods:**

LGs were obtained from adult female rabbits with IAD and age-matched female control rabbits. The LGs were processed for laser capture microdissection (LCM), real time RT–PCR, western blot, and immunofluorescence for the detection of mRNA and proteins of the α1, α2, β1, β2, and β3 subunits of Na^+^/K^+^-ATPase.

**Results:**

In the rabbits with IAD, mRNA levels of α1, β1, and β3 from whole LGs were significantly lower. In samples of acini and epithelial cells from various duct segments, collected by LCM, mRNA levels of α1, β1, β2, and β3 were significantly lower in the rabbits with IAD, although mRNA for α2 could not be detected. However, western blots demonstrated that all five subunits were significantly higher in the rabbits with IAD, although their distribution patterns were similar to those of the control rabbits, as demonstrated by immunofluorescence.

**Conclusions:**

The data presented herein demonstrated significant changes in mRNA and protein expressions of Na^+^/K^+^-ATPase subunits in rabbits with IAD, suggesting that these changes may play a role in the pathogenesis of Sjögren’s syndrome and altered LG secretion, as observed in these animals.

## Introduction

Sjögren’s syndrome is an autoimmune disease that causes functional deficiency of the lacrimal and salivary glands; it is one of the most common causes of dry eye [1]. Although many efforts have been undertaken to understand this debilitating disease, little is known about its etiology [2].

Among many animal models that have been used to study Sjögren’s syndrome, rabbits with induced autoimmune dacryoadenitis (IAD) have been shown to demonstrate many of the ocular surface symptoms and lacrimal gland (LG) pathologies characteristic of Sjögren’s syndrome, and they have been used extensively to study its pathophysiology [3-5].

Like other exocrine gland secretions, LG fluid secretion is an osmotic process mediated by many ion transporters, channels, and aquaporins [6-13]. It is believed that LG fluid secretion is produced in two stages: 1) secretion of primary fluid in the acini and 2) modification into the final fluid during its transit through the duct system. Recent investigations have indicated that the LG ducts also play critical roles in LG fluid production by secreting themselves and/or reabsorbing primary LG fluid [6,7,11,13,14].

Na^+^/K^+^-ATPase, an enzyme located in the plasma membranes of all animals, has been detected in the LGs of rabbits [6,13,15-22] and rats [11,16,23-26], and it has been shown to play a significant role in LG function [6,22]. Na^+^/K^+^-ATPase is a heterotetramer that comprises two α subunits and two β subunits, of which two α subunit isoforms (α1 and α2) and three β subunit isoforms (β1, β2, and β3) have been identified so far, with the β subunit being essential for its normal function [27]. Na^+^/K^+^-ATPase uses the energy released from ATP hydrolysis to move three Na^+^ out of and two K^+^ into the cells, both against electrochemical potential gradients. Depending on the asymmetric localizations of other transport proteins, Na^+^/K^+^-ATPase can power either net absorption or secretion [27].

Although some studies have investigated the role that Na^+^/K^+^-ATPase may play in LG function, little is known about the expression patterns of its subunits in rabbit LG and its potential contribution to LG deficiency in Sjögren’s syndrome. A recent study showed that Na^+^/K^+^-ATPase was one of the main targets of immunoglobulin G (IgG) autoantibodies that interact with the subtype 3 of muscarinic acetylcholine receptors (M3 AChR) in the salivary glands of patients with primary Sjögren’s syndrome, and it was suggested that it may play a role in the pathogenesis of dry mouth [28]. Therefore, the aim of the present study was to investigate the expression patterns of Na^+^/K^+^-ATPase subunits and their potential changes in rabbits with IAD.

## Methods

### Animals and generation of IAD model

Two groups of adult female New Zealand White rabbits (Irish Farms, Norco, CA) were used. One group consisted of six rabbits with IAD, and the other consisted of six age- and sex-matched normal controls. The rabbits were narcotized with a mixture of ketamine (40 mg/ml) and xylazine (10 mg/ml) and given an overdose of Nembutal (80 mg/kg) for euthanasia. Inferior LGs were removed and embedded in OCT, frozen in liquid nitrogen, and stored at −80 °C until use. This study conformed to the standards and procedures for the proper care and use of animals by the US Public Health Service Policy on Humane Care and Use of Laboratory Animals.

To generate the IAD model, the epithelial cells from one LG were first co-cultured with the same rabbit’s ex vivo-activated lymphocytes for two days, and then the cell mixture was injected into the same rabbit’s remaining LG. Typically, the rabbits fully developed IAD within two weeks. The detailed procedures have been published previously [3-5].

### Laser capture microdissection (LCM)

Frozen sections were collected with PEN membrane-coated slides (Leica Microsystems, Buffalo Grove, IL) and stained with cresyl violet in RNase-free conditions with an LCM Staining Kit (Applied Biosystems, Foster City, CA). Acinar cells and epithelial cells from various duct segments were then laser captured using a PixCell II LCM System (Arcturus Bioscience, Mountain View, CA). Approximately 100 cells were collected for each sample for isolation of total mRNA, and six replicates of each acinus and duct segment were collected from each animal [13].

### RNA extraction and reverse transcription

Total cellular RNA was isolated from RNAlater-treated samples with an RNeasy^®^ Midi Kit (Qiagen,Valencia, CA) plus on-column DNase digestion. Detailed procedures were described in our previous report [13]. Briefly, RNA samples were then treated with DNase I to degrade any contaminating DNA and evaluated with a spectrophotometer (ND-1000; Nanodrop Technologies, Wilmington, DE) for quality and quantity. These samples were reverse-transcribed to cDNA with High Capacity cDNA Reverse Transcription Kit with RNase Inhibitor (Applied Biosystems, Foster City, CA) in a thermal cycler (DNA Engine, Bio-Rad, Hercules, CA).

### Real-time RT–PCR analysis and pre-amplification

The sequences of primers and probes used in this study are listed in Table 1. The sequences were selected on computer (Primer Express; ABI) and synthesized by ABI. All probes incorporated the 5′ reporter dye 6-carboxyfluorescin (FAM) and the 3′ quencher dye 6-carboxytertramethylrhodamine (TAMRA). For LCM samples, pre-amplification was performed using a TaqMan® PreAmp Master Mix Kit (Applied Biosystems). The pooled assay mix was prepared by combining up to 50 of 20× gene expression assays into a single tube and subsequently diluted to a final concentration of 0.2×. The 50 μl of pre-amplification reaction included 25 μl of 2× MasterMix, 12.5 μl of 0.2× pooled assay mix, and 12.5 μl of cDNA sample. The reactions were then incubated in the thermal cycler for 10 min at 95 °C followed by 14 cycles at 95 °C for 15 s and 4 min at 60 °C and then held at 4 °C. The pre-amplification product was then diluted 20× with 1× TE (Tris-EDTA, pH 8.0) buffer and analyzed by real-time RT-PCR (TaqMan; Applied Biosystems).

**Table 1:** Primers and probes used for real-time RT-PCR.

**Gene**	**Forward Primer**	**T_m_ (°C)**	**Reverse Primer**	**T_m _(°C)**	**Product Size (bp)**	**Probe**	**Accession #**
*NKAα1*	5'-GCTGTCCATTCATAAGAACCTCAA-3'	53.13	5'-TCTGGAGCGCCCTTCATC-3'	53.35	69	5'-CAATGAGCCACGGCACCTGCTAG-3'	AF235024
*NKAα2*	5'-TCGGGACGGACCCAATG-3'	52.87	5'-AAGCTGGCGACAGAACTTGAC-3'	55.36	70	5'-CCTCACTCCACCCCCGACAACTCC-3'	AF235025
*NKAβ1*	5'-GCCCAGAAGGATGACATGGT-3'	53.83	5'-CGCCTCGTTCTTTAGGTTCACT-3'	54.80	67	5'-TTGAAGATTGTGGCGACGTGC-3'	AF204927
*NKAβ2*	5'-GCCCCGGGCGCTATT-3'	53.38	5'-AGCGCGTTTCGGGTAGTTG-3'	55.40	59	5'-CGAACAGCCGGATAACGGAGTCC-3'	AY069937
*NKAβ3*	5'-TGCTTCAAGCATGCAGTGGTA-3'	54.66	5'-CACAAGAACACAAGGGCTTCCT-3'	55.09	71	5'-AGTGATCCTGATTTTGGCTATTCC-3'	AF302929

For the real-time RT-PCR step, amplification was performed on a sequence-detection system (Prism 7900HT, with TaqMan Gene Expression Master Mix; ABI) containing the internal dye ROX as a passive reference, in accordance with the procedures described. The PCR reaction volume was 10 μl. It contained 1× master mix, 900 nM forward and reverse primers, 250 nM probes, and 2.5 μl of 1× TE–diluted cDNA template. The FAM signal was measured against the ROX signal to normalize for non-PCR-related fluorescence fluctuations. The cycle threshold (CT) value represented the refraction cycle number at which a positive amplification reaction was measured and was set at 10× the standard deviation from the mean baseline emission calculated for PCR cycles 3 through 15. Each sample was measured in triplicate. The difference between the CT for each target mRNA and the internal housekeeping gene glyceraldehyde 3-phosphate dehydrogenase (*GAPDH*) in each sample was used to calculate the level of target mRNA relative to that of *GAPDH* mRNA in the same sample.

### Immunofluorescence and microscopy

The primary antibodies used were all purchased from Santa Cruz Biotechnology (Santa Cruz, CA). The dilution for α1 (mouse monoclonal, sc-71638) was 1:400; for α2 (goat polyclonal, sc-31391), 1:400; for β1 (mouse monoclonal, sc-21713), 1:100; for β2 (mouse monoclonal, sc-135997), 1:100; and for β3 (goat polyclonal, sc-66343), 1:250. Secondary antibodies used were fluorescein isothiocyanate (FITC)-conjugated AffiniPure donkey anti-goat and anti-mouse IgG (Jackson ImmunoResearch Laboratories, West Grove, PA), at a dilution of 1:200. Rhodamine conjugated phalloidin (Invitrogen, Carlsbad, CA), at a dilution of 1:200, was also used to stain F-actin to show the morphological profiles of the LGs.

The samples frozen in Optimal Cutting Temperature (OCT) compound were cut 8 μm thick and placed on slides then fixed with ready-to-use formaldehyde/zinc fixative (Electron Microscopy Sciences, Hatfield, PA) for 15 min. They were then washed in phosphate-buffered solution (PBS) 3× for 10 min each and blocked with donkey normal serum (Jackson ImmunoResearch Laboratories) for 1 h at room temperature. The slides were then incubated with primary antibodies at respective dilutions overnight at 4 °C. On the next day, slides were again washed 3× for 10 min in PBS and incubated with secondary antibody for 1 h at room temperature then washed 3× for 10 min in PBS and 1× for 15 min in 4 mM sodium bicarbonate. Finally, one drop of aqueous mounting medium (Vector Laboratories, Burlingame, CA) was placed on slides and covered with coverslips. Slides were observed with a Leica epifluorescence microscope (Leica Microsystems, Buffalo Grove, IL) and a Zeiss LSM 710 confocal laser scanning microscope (Carl Zeiss Microimaging, Thornwood, NY). FITC-conjugated secondary antibodies were visualized by excitation at 488 nm using an argon laser. Images were analyzed with LSM image browser and PhotoShop (Adobe Systems, Mountain View, CA).

### Western blot

LGs were homogenized in RIPA buffer (50 mM Tris-HCl pH 7.4, 150 mM NaCl, 1 mM EDTA, 1% Triton X 100, 1% Na deoxycholate, 0.1% SDS, 1 mM PMSF, 1 µg/ml aprotinin, 1 µg/ml leupeptin) and centrifuged at 2,000× g for 20 min. The supernatant was denatured in SDS–PAGE sample buffer for 20 min at 60 °C, resolved on a 7.5% or 4%–20% gradient SDS–PAGE gel (Bio-Rad, Hercules, CA), and then transferred onto PVDF membrane (Immobilon-P; Millipore, Billerica, MA). To assess transporter proteins, a constant quantity of proteins from each sample was analyzed. Membrane blots were probed with α1 at a dilution of 1:1,000, α2 at 1:500, β1 at 1:2,000, and β2 and β3 at 1:500. All blots were incubated with Alexa 680-labeled donkey anti-goat or goat anti-mouse secondary antibody (Invitrogen) and detected with an Odyssey Infrared Imaging System (Li-Cor, Lincoln, NE). Densitometry analysis of the resulting gel was performed by the manufacturer’s software.

### Statistics

Data were presented as mean±SEM. Student’s *t*-test and ANOVA were used to evaluate the significance of the differences; a p<0.05 was considered significant.

## Results

### Expressions of mRNA

#### α1

**Figure 1:**
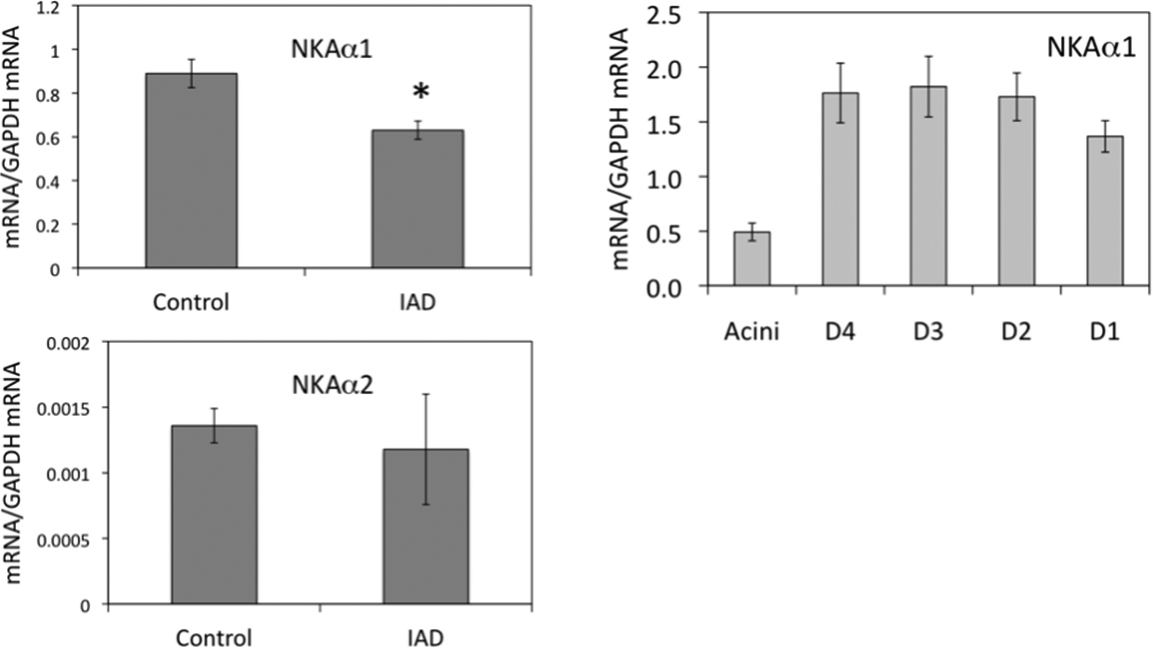
Real-time RT–PCR of α1 and α2 from whole LG (left panels) and lacrimal epithelial cells collected by LCM (right panel). mRNA levels of α1 from whole LG of IAD rabbits were significantly lower than those of control animals (p<0.05), whereas no significant difference was detected between control and IAD animals for α2 (p>0.05). In epithelial cells collected by LCM, mRNA of α1 was the least abundant in acini, whereas its level was significantly higher in all duct segments. Compared to control animals [13], mRNA level of α1 was significantly lower in acini and every duct segment except in intralobar duct in IAD animals (p<0.05). However, we were unable to detect any NKAα2 in epithelial cells collected by LCM due to its low level. D4: intralobular duct. D3: interlobular duct. D2: intralobar duct. D1: interlobar duct. Data were presented as mean±SEM.

The mRNA levels for α1 from whole LG were significantly lower in the animals with IAD (0.63±0.04) compared with the control group (0.89±0.06), with a 29.1% difference (p<0.05; Figure 1). Data from the LCM samples indicated that mRNA for α1 from the rabbits with IAD was least abundant in the acini, consistent with results from normal control rabbits in our previous report [13], and its level was significantly lower in the acini and every duct segment, except the intralobar duct (p<0.05; Table 2).

**Table 2:** mRNA changes from rabbits with IAD compared to controls [13].

**Subunit**	**Whole LG**	**Acini**	**Intralobular**	**Interlobular**	**Intralobar**	**Interlobar**
α1	↓	↓	↓	↓	−	↓
α2	−	ND	ND	ND	ND	ND
β1	↓	↓	↓	↓	↓	↓
β2	−	↓	↓	↓	−	↓
β3	↓	↓	−	−	−	−

Note: ↓: significant decrease; ↑: significant increase; −: no significant difference; ND: not detected.

#### α2

**Figure 2:**
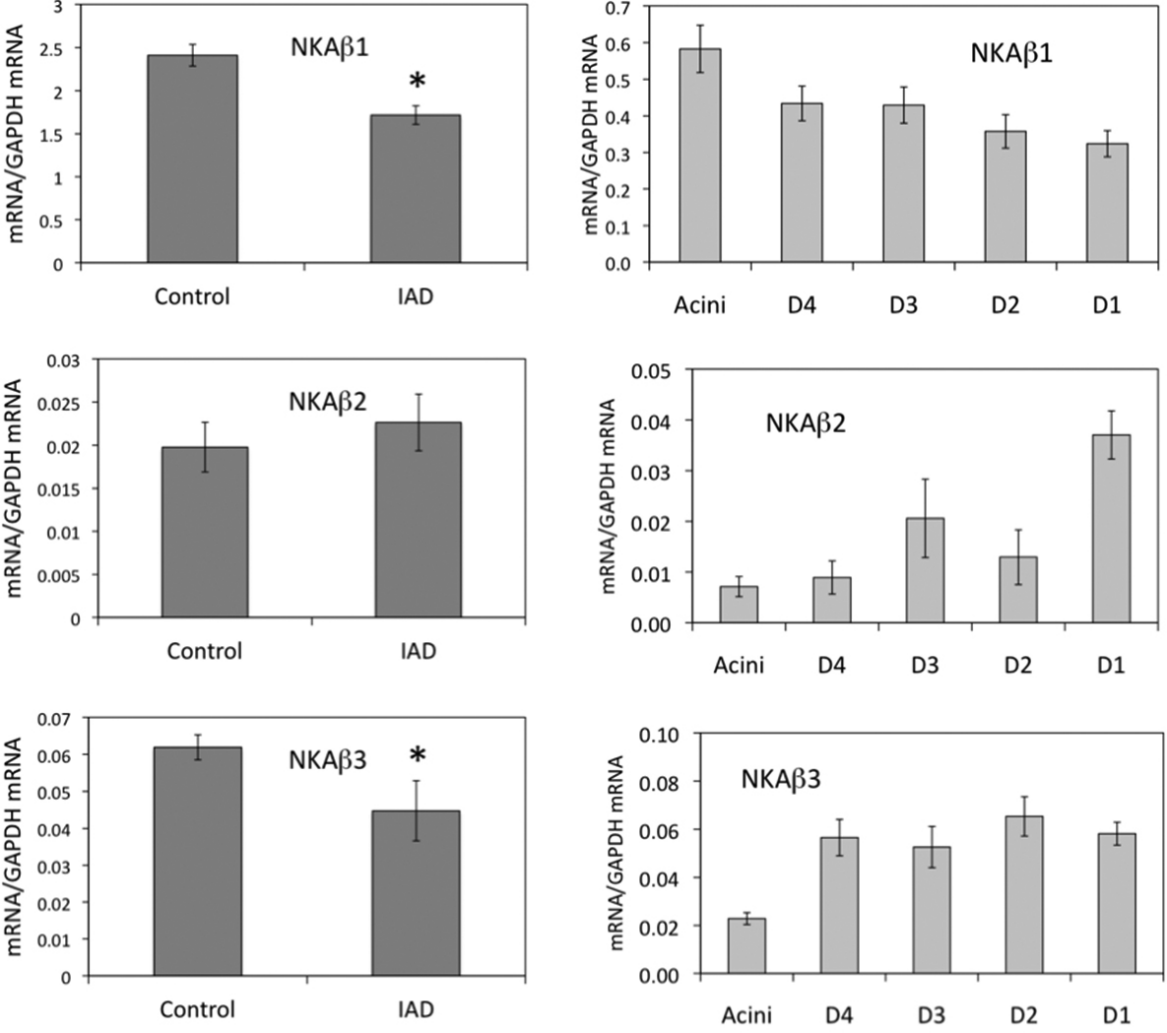
Real-time RT–PCR of β subunits from whole LG (left panels) and epithelial cells collected by LCM (right panels). mRNA levels of β1 and β3 from whole LG of IAD rabbits were significantly lower than control animals (p<0.05), whereas no significant difference was detected between control and IAD animals for β2 (p>0.05). β1: In epithelial cells collected by LCM, β1 was most abundant in acini, and their level in IAD appeared to be similar in duct segments and was significantly lower than control animals [13] in acini and all duct segments (p<0.05). β2: mRNA was least abundant in acini and most abundant in interlobar duct, and its levels in IAD rabbits were significantly lower than control animals in acini and all duct segments except in intralobar duct (p<0.05). β3: mRNA was least abundant in acini while its levels were similar in all duct segments. Compared to control animals [13], its levels in IAD animals were significantly lower in acini (p<0.05), while no differences were detected in duct segments (p>0.05). D4: intralobular duct. D3: interlobular duct. D2: intralobar duct. D1: interlobar duct. Data were presented as mean±SEM.

The expression of mRNA for α2 was very low in the LGs; in fact, it was the least abundant of all Na^+^/K^+^-ATPase subunits (Figure 1 and Figure 2). No significant difference of α2 mRNA from whole LG was detected (p>0.05) between the rabbits with IAD (0.0012±0.0004) and the control animals (0.0014±0.0001), and we were unable to detect any presence of α2 mRNA in the epithelial cells collected by LCM.

#### β1

mRNA for β1 from whole LG was significantly lower in the animals with IAD (1.717±0.107) than in the control group (2.411±0.125), with a 28.8% difference (p<0.05; Figure 2). Data from the LCM samples showed that mRNA for β1 was most abundant in the acini, and its levels were significantly lower than in the control animals [13] in the acini and all duct segments (p<0.05).

#### β2

No significant difference of β2 mRNA from whole LG was detected (p>0.05) between the control rabbits (0.02±0.003) and the rabbits with IAD (0.023±0.003; Figure 2). mRNA from the epithelial cells collected by LCM was least abundant in the acini and most abundant in the interlobar duct, and its levels were significantly lower than in the control animals [13] in the acini and all duct segments, except in the intralobar duct (p<0.05).

#### β3

mRNA for β3 from whole LG was significantly lower in the animals with IAD (0.045±0.008) than in the control group (0.062±0.003), with a 27.7% difference (p<0.05; Figure 2). Data from the LCM samples showed that mRNA for β3 was least abundant in the acini, and its levels were significantly lower in the acini (p<0.05), while no differences were detected in the duct segments (p>0.05) compared to the control animals [13].

### Western blot and densitometry

**Figure 3:**
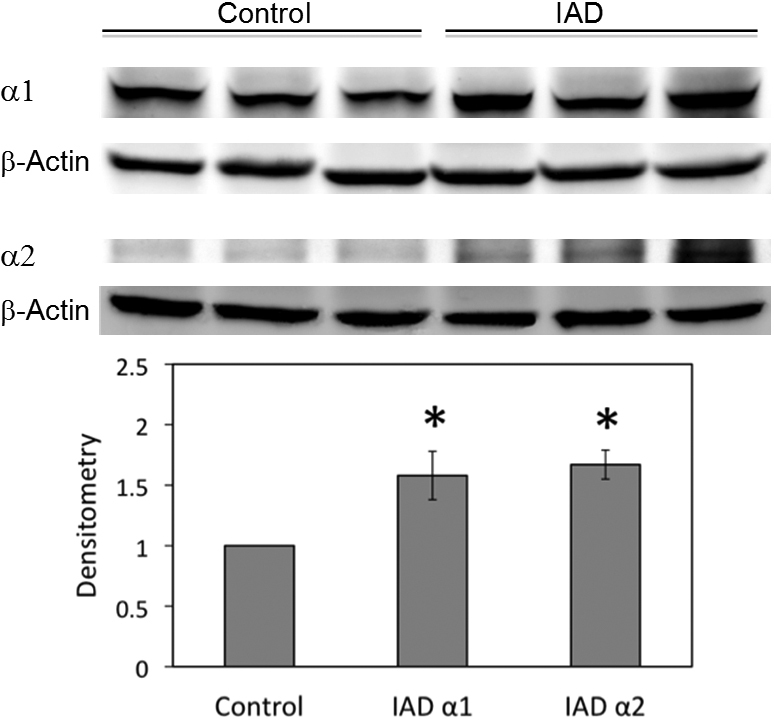
Western blots of α subunits from whole LG homogenates. Both α1 and α2 were significantly increased in LG from rabbits with IAD (p<0.05). β-Actin was used as loading controls. Data are representative images of at least 3 different animals each.

**Figure 4:**
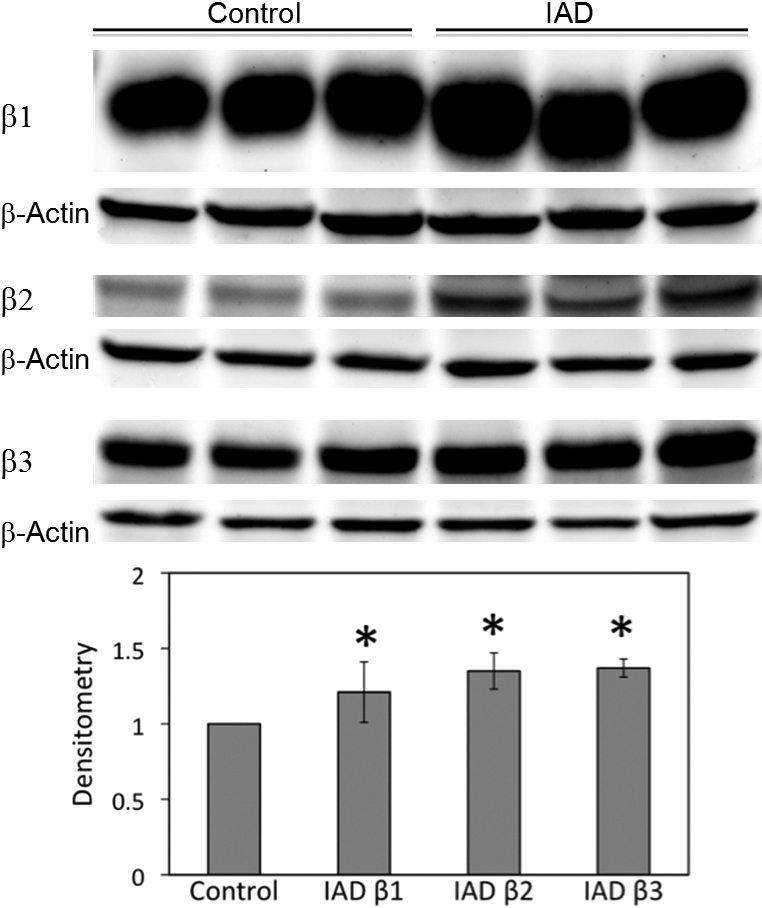
Western blots of β subunits from whole LG homogenates. All β subunits were significantly increased in LG from rabbits with IAD (p<0.05). β-Actin was used as loading controls. Data are representative images of at least 3 different animals each.

We studied the expressions of α (Figure 3) and β (Figure 4) subunits by the immunoblotting of whole LG homogenates. Densitometry analysis showed that expressions of α1 from the rabbits with IAD were 58% higher and expressions of α2 were 67% higher than the control rabbits, both significantly different (p<0.05). The expressions of all three β subunits were significantly higher in the rabbits with IAD, with differences of 21% for β1, 35% for β2, and 37% for β3 (p<0.05).

### Immunofluorescence

#### α1

**Figure 5:**
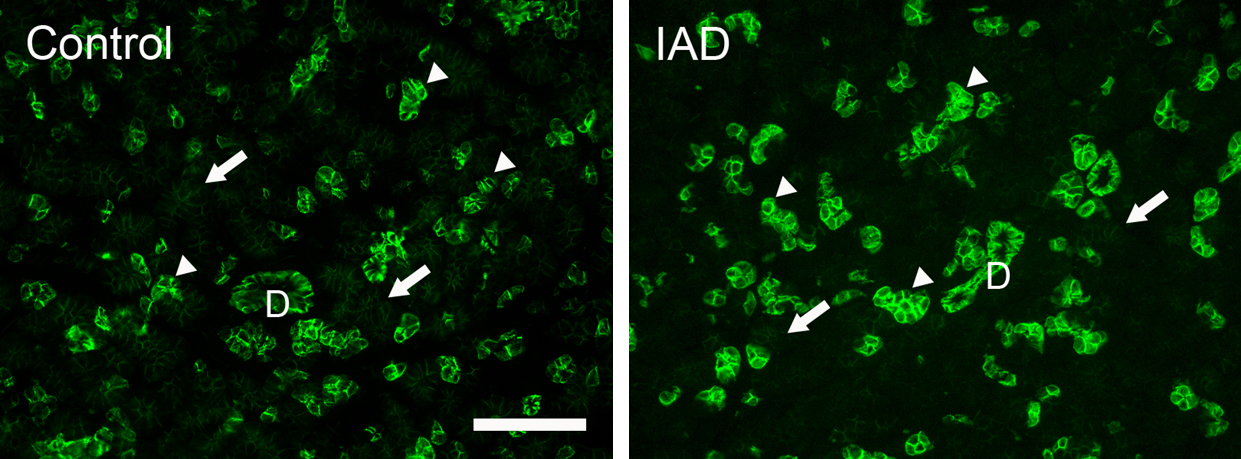
Immunofluorescence of α1. Control: α1-IR (green) was present in every acinar cell, most prominently in the basolateral membranes, but not on the apical membranes. The α1-IR was much stronger in some acini/acinar cells (arrowheads), while much weaker in other acini/acinar cells (arrows), giving the gland a “mosaic” pattern. Ducts (D) were all stained as strongly as those acinar cells showing intense α1-IR. IAD: the distribution pattern of α1-IR was similar to those in control rabbits, and no significant difference was detected. Scale bar=100 μm.

Na^+^/K^+^-ATPase α1 immunoreactivity (IR) was detected in all acinar cells, most prominently on the basolateral membranes (Figure 5). No α1-IR was found on the apical membranes. However, the α1-IR showed a “mosaic” pattern among the acini, i.e., some acini/acinar cells showed much stronger α1-IR, while the intensity in other acini was much weaker. The ducts were all stained as intensely as the acinar cells showing the most intense α1-IR. In LGs from the rabbits with IAD, the distribution pattern of α1-IR was similar to those in the control rabbits, and no significant difference was detected.

#### α2

**Figure 6:**
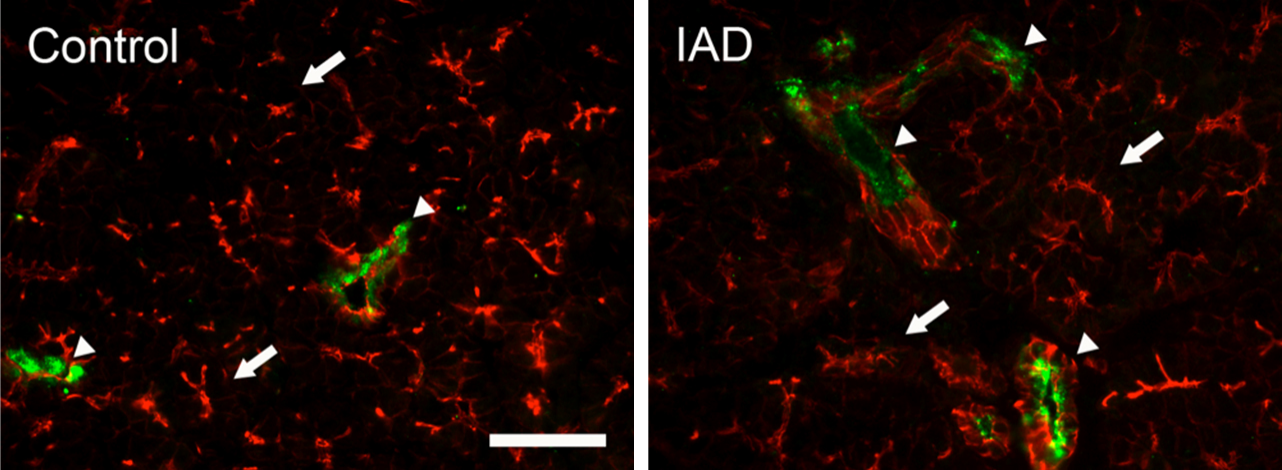
Immunofluorescence of α2. Control: Minimal α2-IR was detected in acinar cells (arrows), while strong α2-IR was observed in ductal cells as clustered punctate staining (arrowheads). IAD: The distribution of α2-IR in LG from rabbits with IAD was very similar to that of control animals, i.e., α2-IR was mostly found in ductal cells as clusters of punctate staining. Red: F-actin that has been stained with rhodamine-conjugated phalloidin. Scale bar=50 μm.

In LGs from the control animals, minimal α2-IR was detected in the acinar cells, while its intensity was much stronger in the ducts and appeared as clustered punctate staining (Figure 6). The distribution of α2-IR in LGs from the rabbits with IAD was very similar to that of the control animals, and no significant difference was observed.

#### β1

**Figure 7:**
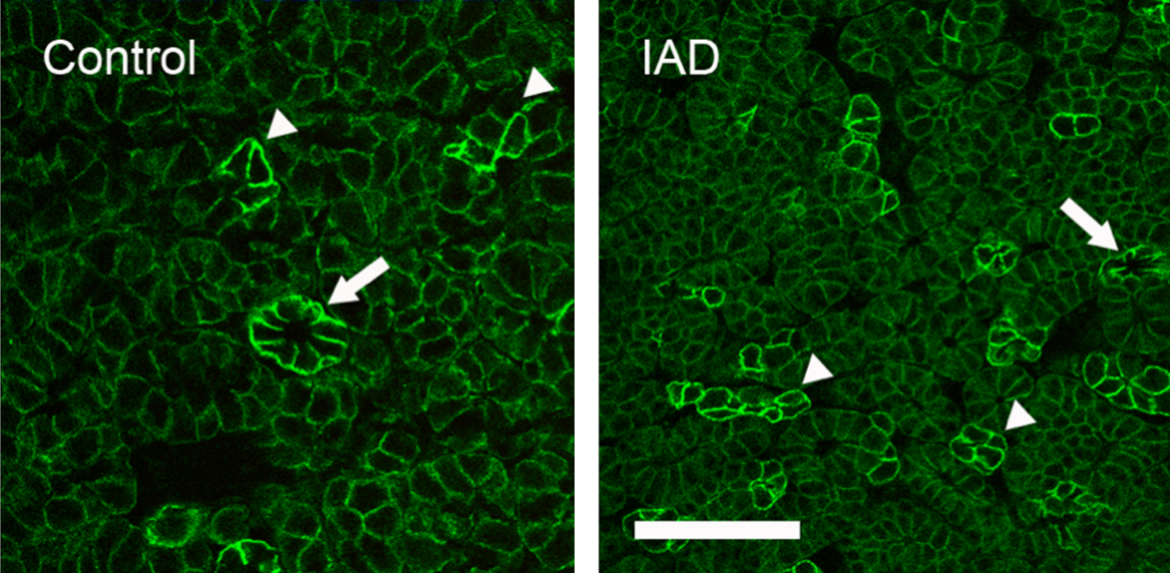
Immunofluorescence of β1. Control: Like α1, β1-IR was also only present in the basolateral membranes of all acinar and ductal cells, and numerous punctate staining within the cytoplasm. However, levels of β1-IR differed in a “mosaic” pattern, higher in some acinar cells and/or acini (arrowheads) than others. β1-IR in ductal cells was uniformly higher (arrow). These data were similar to our previous report [13]. IAD: like in control animals, β1-IR was also present in all acinar cells and demonstrated a similar “mosaic” pattern (arrowheads). Ducts also showed a much higher β1-IR (arrow). No significant difference of β1-IR was observed between control and IAD animals. Scale bar=50 μm.

Similar to the pattern of α1, β1-IR was also only detected in the basolateral membranes of all acinar and ductal cells, but not on the apical membranes (Figure 7). Numerous punctate staining was also present within the cytoplasm. In addition, levels of β1-IR differed in a “mosaic” pattern, higher in some acinar cells and/or acini than in other acini, a pattern similar to that of α1. β1-IR in the ductal cells was much stronger. These results were consistent with our previous report [13]. In the rabbits with IAD, β1-IR was also present in all acinar cells and demonstrated a “mosaic” pattern similar to that observed in the control animals. The ducts also showed much higher β1-IR levels. No significant difference was observed between the control and IAD animals.

#### β2

**Figure 8:**
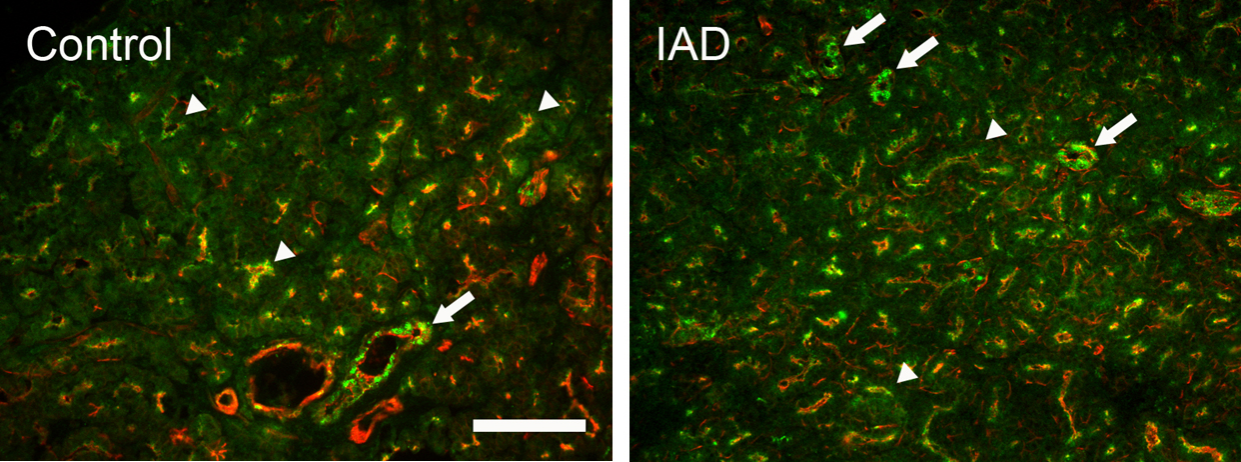
Immunofluorescence of β2. Control: β2-IR (green) was present in all acinar cells as numerous punctate staining that aggregate toward the apical cytoplasm (arrowheads), while its intensity was much higher in ducts (arrow). IAD: the distribution pattern of β2-IR was similar in LG from rabbits with IAD with those in control rabbits, i.e., numerous punctate β2-IR was detected in the cytoplasm of every acinar cell (arrowheads), while β2-IR was much stronger in ducts (arrows). Red: F-actin that has been stained with rhodamine-conjugated phalloidin. Scale bar=100 μm.

β2-IR was detected in all acinar cells as numerous punctate staining that aggregated toward the apical cytoplasm (Figure 8); its intensity was much higher in the ducts. The distribution pattern of β2-IR from the rabbits with IAD was similar to that of the LGs from the control rabbits, and no significant difference was observed.

#### β3

**Figure 9:**
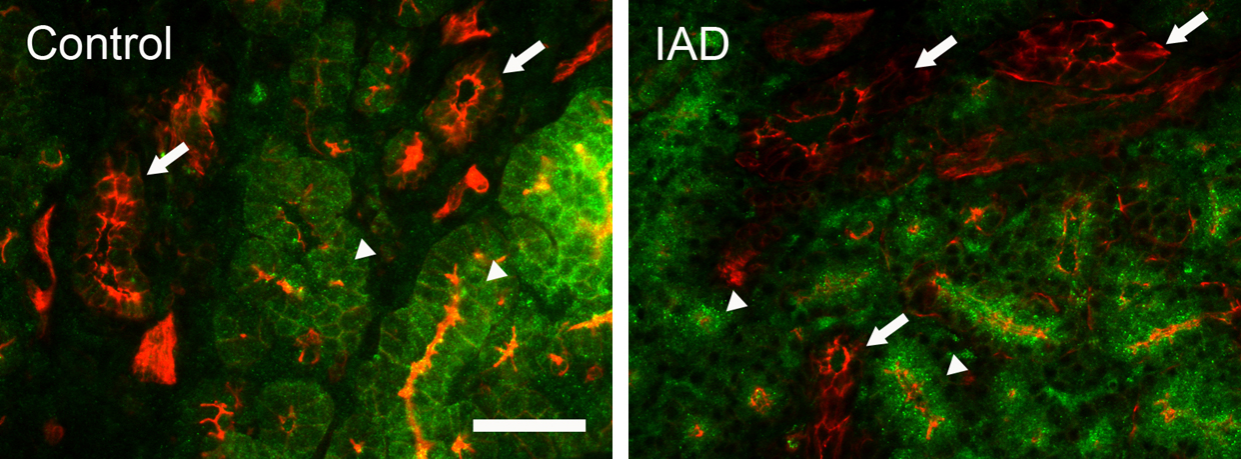
Immunofluorescence of β3. Control: β3-IR (green) was present in every acinar cell as numerous punctate staining within the cytoplasm (arrowheads) in a diffuse pattern. However, minimal β3-IR was detected in the duct cells (arrows). IAD: the distribution pattern of β3-IR from rabbits with IAD was similar to that of control rabbits, i.e., numerous punctate β3-IR was detected in the cytoplasm of every acinar cell (arrowheads), while minimal β3-IR was present in the duct cells (arrows). Red: F-actin that has been stained with rhodamine. Scale bar=50 μm.

β3-IR was detected in all acinar cells in a diffuse pattern as numerous punctate staining within the cytoplasm, while minimal β3-IR was detected in the ducts (Figure 9). In the rabbits with IAD, the distribution pattern of β3-IR was similar to that of the control rabbits.

## Discussion

Our real-time RT–PCR data demonstrated the presence of all five subunits of Na^+^/K^+^-ATPase in the rabbit LGs, although their levels varied greatly. α1 was the dominant α subunit, with an abundance approximately 650 times that of α2. In fact, α2 levels were so low that it was undetectable in the LCM samples. Like α1, β1 was also the dominant β subunit, with its mRNA levels approximately 120 times that of β2, and 39 times that of β3. These data appear to suggest that α1β1 is the dominant heterodimer, which is supported by our immunofluorescence results. Both α1 and β1 were present in the basolateral membranes in a “mosaic” pattern in the LGs (Figure 5 and Figure 7), i.e., they were particularly rich in some acini and/or acinar cells, while the rest of the acini demonstrated much weaker intensity. The ductal cells also demonstrated intense staining with both α1 and β1.

Our recent results showed that the acini and/or acinar cells that displayed intense β1 staining were in fact mucous cells capable of secreting mucins, along with all ductal cells. The results clearly demonstrate that the rabbit LG is a mixed gland comprised of both serous and mucous cells, and it also contributes to the mucin pool in the tear film [29].

α2 was detected by immunofluorescence only in the ductal cells. However, while particularly rich in the ducts, β2 was also found in apical cytoplasm of all acinar cells as numerous punctate staining. Interestingly, β3 was only detected in the acini and was virtually absent from the ducts.

Our immunofluorescence results appear to be different from those reported by Bradley et al. [18], who showed that antibodies against the Na^+^/K^+^-ATPase holoenzyme and α1 were detected in both apical and basolateral membranes of the acinar cells from rat LG, although the intensity to α1 was relatively weaker, particularly in the apical membranes. In addition, intense punctate staining was detected in the apical cytoplasm of both antibodies, and the ductal cells were positively stained. β1 staining was mostly observed as a diffuse cytoplasmic distribution in the acinar cells, with some focal concentrations.

Other studies have demonstrated the presence of Na^+^/K^+^-ATPase in both acinar and ductal cells from LGs of rats [11,16,23-26] and rabbits [6,13,15-22]. However, another study, on mouse and rat LGs, showed that the acinar cells failed to be stained with Na^+^/K^+^-ATPase, while all segments of the duct system stained intensely in the basolateral membranes [30].

Dartt et al. [6], in a pioneering and elegant study, first demonstrated the presence of Na^+^/K^+^-ATPase in the LG and showed that the ductal cells were actively involved in lacrimal secretion, which supports the idea of Na^+^/K^+^-ATPase as a major factor in LG function. The authors also suggested that the cholinergic activation of LG secretion is dependent upon Na^+^/K^+^-ATPase, which derives energy from ATP hydrolysis to drive the efflux of Na^+^ and influx of K+ through the plasma membranes, both against electrochemical gradients. The resulting Na^+^ gradient provides the direct energy for other transporters and channels, a notion that has been supported by inhibitory studies that showed that ouabain, the specific inhibitor of Na^+^/K^+^-ATPase, inhibited rabbit LG secretion [31] and completely abolished carbachol-induced short-circuit currents in a rabbit acinar cell monolayer cultured on polyester membrane scaffolds [22].

It has been shown that Na^+^/K^+^-ATPase expression undergoes significant changes under several conditions. LGs of female rat showed rapid atrophy after hypophysectomy, and total Na^+^/K^+^-ATPase activity was reduced by half, while dihydrotestosterone (DHT) and prolactin treatment both partially restored its activity [26]. However, DHT treatment of ovariectomized rabbits increased Na^+^/K^+^-ATPase activity by 29%, while the synthetic estrogen diethylstilbestrol (DES) decreased total Na^+^/K^+^-ATPase by 12% [20].

In rat LGs, cholinergic stimulation caused translocation of Na^+^/K^+^-ATPase from intracellular pools to basolateral membranes [16]. Carbachol stimulation of primary cultured rabbit LG acinar cells also caused a significant decrease of total content of Na^+^/K^+^-ATPase, suggestive of its increased flux to lysosomes, where it was degraded [21]. Cholinergic stimulation of rabbit acinar cells demonstrated that Na^+^/K^+^-ATPase cytoplasmic reserves were recruited to the basolateral membrane [15-17], suggesting that the intracellular reserves of Na^+^/K^+^-ATPase are available for rapid recruitment to the basolateral membranes. It has also been suggested that the carbachol-induced redistribution of Na^+^/K^+^-ATPase to the basolateral membrane represents a mechanism by which the cell compensates for the increased Na^+^ influx [16].

The only literature regarding the presence of Na^+^/K^+^-ATPase in the LGs of Sjögren’s syndrome patients showed that the distribution of Na^+^/K^+^-ATPase was unchanged in the LGs, although its expression was not quantified [32]. However, a recent study on salivary glands, another exocrine gland that is very similar to LG anatomically and physiologically, showed that in salivary glands from patients with primary Sjögren’s syndrome, Na^+^/K^+^-ATPase was one of the main targets of anti-M3 IgG. These patients also produced functional IgG autoantibodies that acted as partial muscarinic agonists in the submandibular gland and increased PGE2 and cAMP production, therefore inhibiting Na^+^/K^+^-ATPase activity. These autoantibodies could also interfere with the secretory effect of parasympathetic neurotransmitters, and may play a role in Sjögren’s syndrome-related dry mouth [28].

In LG samples from rabbits with IAD, the mRNA levels from whole LG of α1, β1, and β3 were significantly lower than those of the control animals, while the levels of α2 and β2 remained unchanged. Compared to the results from the control rabbits [13], mRNA levels were significantly lower in many duct segments (Table 2). However, our western blots showed opposite results, i.e., the protein expressions of all subunits were significantly higher in the rabbits with IAD, although none of the subunits’ distribution patterns showed noticeable differences. Whether the increased expressions of Na^+^/K^+^-ATPase subunits in rabbits with IAD was a primary or secondary consequence of IAD remains unknown, but given the fact that LG fluid production in these animals was greatly reduced [3-5], it is more likely that the increased protein expressions were due to compensation for reduced LG secretion.

It should be noted that there were discrepancies in mRNA and protein expressions in the data presented herein, which also have been reported in previous studies in other tissues and organs [33,34]. Various mechanisms could be responsible for these discrepancies; changes in protein redistribution and recycling during inflammation, such as IAD, may be possible reasons [35,22]. Furthermore, differences in protein expression may also not always reflect the functional status in the phenotype. However, this topic is beyond the scope of the present study but highlights the importance of direct functional studies of the LG’s functional changes in rabbits with IAD to provide definitive evidence.

Despite the fact that up to 15% of all epithelial cells in the LG are ductal cells [36-38], most of the previous LG studies have focused on the acinar cells, which represent approximately 80% of all epithelial cells, with only a few studies having paid sufficient attention to the duct system [6,11,13,14,29,39]. The significant presence of Na^+^/K^+^-ATPase in the ductal cells, and their significant changes in rabbits with IAD, point to active Na^+^ and K^+^ transport in the ductal cells and their potential contribution to LG deficiency during IAD.

In summary, our findings demonstrated that there were significant changes in mRNA and protein expression of Na^+^/K^+^-ATPase subunits in the acinar and ductal cells of rabbits with IAD. These data strongly suggest that changes in this solute transporter and its subunits may contribute to reduced tear secretion in rabbits with IAD, although direct functional studies are needed to provide definitive evidence. Data presented herein also support the notion that acini and ducts play different roles in LG secretion. However, the exact mechanisms of how Na^+^/K^+^-ATPase functions in the LG in physiologic and pathological conditions are unknown, and more studies into their roles are certainly needed.
